# Prevalence and antimicrobial susceptibility profile of multidrug-resistant bacteria among intensive care units patients at Ain Shams University Hospitals in Egypt—a retrospective study

**DOI:** 10.1186/s42506-020-00065-8

**Published:** 2021-03-29

**Authors:** Noha Alaa Eldin Fahim

**Affiliations:** Clinical Pathology Department, Faculty of Medicine, Ain Shams University, Cairo, Egypt

**Keywords:** Multidrug resistance; Egypt, Prevalence, ICU, Susceptibility profile

## Abstract

**Background:**

The nightmare of the rising numbers of multidrug-resistant organisms (MDROs) requires the implementation of effective stewardship programs. However, this should be preceeded by making available  evidence-based knowledge regarding the local antimicrobial resistance pattern, which is fundamental. The aim of the current study is to determine the prevalence of MDRO among different Ain Shams University Hospitals (ASUHs) intensive care units (ICUs) and detect the resistance profile of the common pathogens.

**Results:**

The 1-year records of a total of 1280 pathogens were studied. The highest number of pathogens were isolated from blood cultures (44.84%), followed by urine (41.41%) then wound swabs (13.75%). Gram-negative isolates (57.5%) were more prevalent than gram-positive ones (31.1%). The most frequently isolated pathogens were *Klebsiella* spp. (22.5%), *Escherichia coli* (13.4%), and *Coagulase-negative Staphylococci* (12.5%). The highest percentage of resistance among gram-positive organisms was exhibited by penicillin (89.5%) followed by erythromycin (83.98%) and then cefoxitin (76.52%). None of the isolates showed resistance to linezolid and resistance to vancomycin was minimal (2.62%). Gram-negative isolates exhibited high overall resistance to all used antibiotic classes. The least frequency of resistance was recorded against nitrofurantoin (52.5%), amikacin (58.01%), followed by imipenem (59.78%) and meropenem (61.82%). All isolates of *Pseudomonas* and *Acinetobacter* showed 100% susceptibility to colistin.

**Conclusions:**

The prevalence of antibiotic resistance in Ain Shams University Hospitals (ASUHs) was high among both gram-negative and gram-positive organisms. This high resistance pattern foreshadows an inevitable catastrophe that requires continuous monitoring and implementation of effective antibiotic stewardship.

## Background

Antimicrobial resistance is rapidly becoming a global focus of attention, especially with the rising number of microorganisms resistant to available antimicrobials. It encompasses both the gram-positive and gram-negative bacteria, with global prevalence rates of 60% or more [1].

Multidrug-resistant organisms (MDROs) are described as acquired non-sensitivity to one or more agents in at least three groups of antimicrobials. This kind of resistance essentially predominates in hospitals [2].

The lack of quick proper identification of pathogens especially in patients with critical infection led to broad-spectrum antibiotics overuse. As a result of this dilemma, organisms became resistant to all available antimicrobial agents and susceptible only to older, likely more toxic antimicrobials, leaving less effective scanty alternatives [3, 4]. The Centers for Disease Control and Prevention (CDC) declared that worldwide increasing infection rates with resistant pathogens strikingly endanger our healthcare systems creating both negative universal economic effects and a therapeutic challenge for clinicians hence delaying proper antibiotic therapy and increasing mortality rates [5].

To combat this horrifying ascent in antimicrobial resistance, the World Health Organization (WHO) urges healthcare providers to adopt antimicrobial stewardship to decrease the heavy cargo of antibiotic resistance. However, before the implementation of any stewardship program, information on prevalent MDRO and their antimicrobial resistance profile are required [6]*.*

Data about the endemic antimicrobial resistance are generally difficult to find, particularly in countries where antibiotics are easily obtainable over the counter. Although numerous reports demonstrated the incidence and the patterns of resistance of many pathogens, few studies about the endemic antimicrobial resistance profile in developing countries were published [3, 7].

Hence, an evidence-based knowledge regarding the local antimicrobial resistance pattern is fundamental for guiding both antimicrobial treatment and empirical therapy of specific pathogens [8]. This guide is also important for effective antimicrobial stewardship as well as in the design of local and universal research programs [3].

Since the intensive care unit (ICU) patients are more prone to nosocomial infections caused by aggressive pathogens, therefore, the present study aimed to identify and obtain a comprehensive idea about the current situation in Ain Shams University Hospitals (ASUHs) regarding the spectrum of microbes and the antimicrobial resistance pattern of the most prevalent pathogens isolated from variable infection sites of ICU patients in addition to the determination of the prevalence of multiple drug resistance through a 1-year retrospective study.

## Methods

### Study design

A record-based 1-year retrospective study—from March 2018 going through February 2019—was conducted at ASUHs one of the largest tertiary hospitals in Egypt with more than 3200-bed capacity.

The records of the pathogenic organisms recovered from different microbiological samples (blood, urine, and wound) of ICU patients sent for routine diagnosis in microbiology laboratory were retrieved from the microbiology laboratory information system at ASUHs and reviewed. Information regarding the identified bacterial isolate, specimen type, and antimicrobial susceptibility was collected and recorded.

### Microbiological specimens processing and identification of isolated organisms

Sample processing and identification of the microorganism were performed per the standard operating procedures of our laboratory. All the used media were purchased from (Oxoid, UK).

The samples were cultured on the routinely used microbiological media and incubated for 24 h at 37 °C. If no growth, the plates were incubated for a total of 48 hours.

The isolated microorganisms' recognition was done according to colony morphology, Gram stain, and standard confirmatory biochemical tests were used.

Gram-positive bacteria were identified via testing the hemolytic activity on blood agar and further identification using different biochemical tests as catalase reaction, slide and tube coagulase tests, culture on DNase agar, bile esculin, in addition to different differentiating antibiotic discs as optochin and bacitracin. For gram-negative bacteria, identification was conducted by biochemical tests such as oxidase, triple sugar iron, motility indole ornithine, citrate, lysine iron arginine, and urease tests. They were further identified by Vitek2 system (Biomerieux, France).

### Antimicrobial susceptibility

Antimicrobial susceptibilities of the bacterial isolates by Kirby-Bauer disk diffusion method were performed and interpreted according to the Clinical Laboratory Standards Institute (CLSI) guidelines [9]. The tested antibiotic discs were routinely supplied from Oxoid. *Staphylococcus aureus* ATCC 25923, *Pseudomonas aeruginosa* ATCC 27853, and *Escherichia coli* ATCC 25922 were used as controls for susceptibility testing.

Extended-spectrum beta-lactamase (ESBL) production was detected using the double-disc synergy test by placing amoxicillin/clavulanate disc adjacent to cefotaxime and ceftazidime discs and looking for synergy between the clavulanic acid and the cephalosporin [10].

Colistin resistance in both *Acinetobacter* spp*.* and *Pseudomonas* spp. was detected by Vitek2 system.

Methicillin resistance in *Staphylococcus* species both *Staphylococcus aureus* (MRSA) and coagulase-negative (MR-CONS) was detected using cefoxitin disc (30 μg).

Vancomycin-resistant *Enterococci* (VRE) were initially screened for using agar dilution method on brain heart infusion agar (BHI) with 6 μg/ml vancomycin and further confirmation for any suspected colonies was accomplished by Vitek 2 system (Biomerieux, France).

### Multiple drug resistance (MDR)

MDR isolates were described by the CDC as acquired non-sensitivity to one or more agents in at least three groups of antimicrobials [11].

### Statistical analysis

Data were presented as percentages and counts. Statistical analysis was performed using the statistical package for social sciences (SPSS) computer software (Version 25), IBM software, USA. Pearson chi-square test or Fisher’s exact test—after checking the applicability conditions—were performed to identify the significant effect of each antibiotic on different isolates. A statistically significant difference was considered at *p* value ≤ 0.05.

## Results

### Spectrum of pathogens in different clinical specimens

**Fig. 1:**
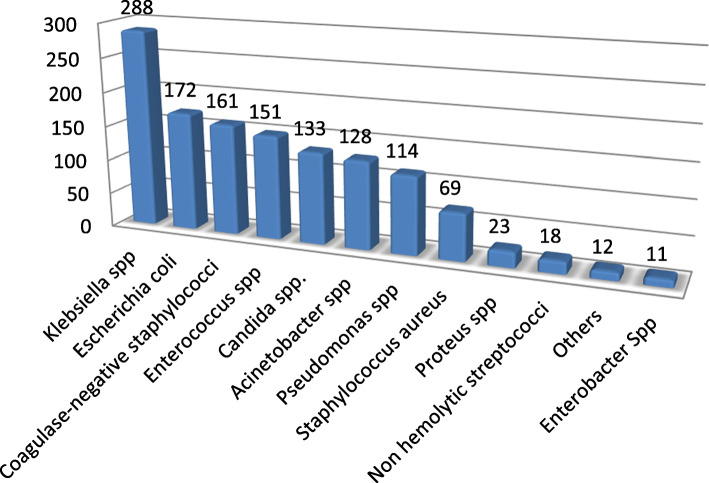
Distribution of different clinical isolates among patients admitted in ICU, March 2018–February 2019

The 1-year records of a total of 1280 pathogens from different clinical samples were retrieved from the laboratory information system of ASUH during the specified time period. The highest number of pathogens were isolated from blood cultures (44.84%, *n* = 574), followed by urine where (41.41%, *n* = 530) pathogens were recovered, while the least isolation rate was exhibited by wound swabs (13.75%, *n* = 176). Gram-negative isolates (57.5%, *n* = 736) were more prevalent compared to gram-positive ones (31.1%, *n* = 399). The most frequently isolated pathogens were *Klebsiella* spp. (22.5%, *n* = 288), *Escherichia coli (E. coli*) (13.4%, *n* = 172), and *Coagulase-negative Staphylococci (CONS)* (12.5%, *n* = 161), while *Proteus*, non-hemolytic *Streptococci*, *Enterobacter* spp., and others constituted the smallest group among the studied isolates (Fig. 1). Bloodstream infections were caused mainly by CONS (24.2%, *n* = 139/574) and *Klebsiella* spp. (23.8%, *n* = 137/574). *Candida* spp. (22.5%, *n* = 126/530), *E. coli* (20%, *n* = 106/530), and *Klebsiella* spp. (19%, *n* = 101/530) were the main incriminated pathogens in urinary tract infections. The frequently isolated pathogens from wounds were *Klebsiella* spp. (28.4%, *n* = 50/176), *Pseudomonas* spp. (17.6%, *n* = 31/176), and *Acinetobacter* spp. (15.9%, *n* = 28/176). The distribution of pathogens among the different types of specimens is summarized in Table 1.

**Table 1:** Frequency of different pathogens within various clinical specimens collected from ICU patients, Ain Shams University Hospitals, Egypt, March 2018–February 2019

Name of organism	Total	***N*** (%)		
		Urine	Blood	Wound swab
***Klebsiella*** **spp.**	288	101 (35.06%)	137 (47.56%)	50 (17.38%)
***E.coli***	172	106 (61.62%)	48 (27.91%)	18 (10.47%)
***Acinetobacter*** **spp.**	128	33 (25.78%)	67 (52.34%)	28 (21.88%)
***Pseudomonas*** **spp.**	114	49 (42.98%)	34 (29.82%)	31 (27.20%)
***Proteus*** **spp.**	23	5 (21.73%)	6 (26.10%)	12 (52.17%)
***Enterobacter*** **spp.**	11	2 (18.18%)	8 (72.72%)	1 (9.10%)
***Coagulase negative staphylococci (CONS)***	161	20 (12.42%)	139 (86.33%)	2 (1.25%)
***Enterococcus*** **spp*.***	151	74 (49.00%)	64 (42.38%)	13 (8.62%)
***Candida*** **spp*.***	133	126 (94.73%)	6 (4.51%)	1 (%0.76)
***Staphylococcus aureus***	69	5 (7.26%)	46 (66.66%)	18 (26.08%)
***Non-hemolytic Streptococci***	18	6 (33.33%)	11 (61.11%)	1 (5.56%)
***Others***	12	3 (25.00%)	8 (66.67%)	1 (8.33%)
**Total**	1280	530 (41.41%)	574 (44.84%)	176 (13.75%)

### Antibiotic resistance pattern of isolates recovered from various sites of infection

The distribution of pathogens and their patterns of resistance are presented in Table 2.

#### Gram-positive isolates

The analysis of the antibiotic susceptibility profile of different gram-positive organisms was conducted and showed that the highest percentage of resistance was exhibited towards penicillin (89.5%) followed by erythromycin (83.98%) and then cefoxitin which is the representative of the different beta-lactams in *Staphylococcus* species (76.52%). On the other hand, linezolid displayed no resistance (0%) and vancomycin resistance was minimal (2.62%) among gram-positive organisms.

 With regards to the predominant resistance phenotypes among different isolates, *Staphylococcus aureus* (*S. aureus*) exhibited high resistance rates to many antibiotics where 97.1% of the isolates displayed resistance to penicillin, and 73.91% were resistant to gentamicin and all beta-lactams. On the other hand, *S. aureus* isolates exhibited a suscepibility of more than 40% to the rest of antibiotics with 100% susceptibility to linezolid, nitrofurantoin, and vancomycin where no vancomycin-intermediate *S. aureus* (VISA) or vancomycin-resistant *S. aureus* (VRSA) were found in this study.

Similarly, CONS showed comparable beta-lactam resistance rates to *S. aureus* with a slightly higher level of methicillin resistance (77.6%), as well as, 100% susceptibility to linezolid and vancomycin. However, CONS displayed a higher level of resistance to the majority of the rest of the antibiotics.

*Enterococci* expressed a high level of resistance to both beta-lactams and quinolones,  ciprofloxacin (86.75%), levofloxacin (76.15%), penicillin (78.14%), and ampicillin (64.9%). In a similar pattern to *Staphylococci*, no linezolid resistance was detected and vancomycin-resistant *Enterococci* (VRE) were discovered in ten isolates (6.62%). Data also revealed a statistically significant difference in the antimicrobial potentials to different isolates. The results of the antibiotic susceptibility profile of each of the Gram-positive pathogens are summarized in Tables 2 and 4.

**Table 2:** Distribution of pathogens associated with intensive care unit-associated infections and their antimicrobial resistance patterns, Ain Shams University Hospitals, Egypt, March 2018–February 2019

Pathogen type and pattern of resistance	***n***	Resistance, ***n*** (%)
***Klebsiella*** **spp.**	288	
ESBL production		30 (10.4)
Multidrug resistance		253 (87.84)
***Acinetobacter*** **spp.**	128	
Multidrug resistance		107 (83.59)
***Pseudomonas*** **spp.**	114	
Multidrug resistance		84 (73.68)
***Escherichia coli***	172	
ESBL production		80 (46.5)
Multidrug resistance		124 (72.02)
***Proteus*** **spp.**	23	
ESBL production		4 (17.4)
***Enterobacter*** **spp.**	11	
ESBL production		4 (36.36)
***Staphylococcus aureus***	69	
MRSA		51 (73.9)
***Coagulase-negative staphylococci***	161	
MR-CONS		125 (77.6)
***Enterococcus*** **spp.**	151	
VRE*		10 (6.6)
***Candida*** **spp.**	133	
***Non-hemolytic streptococci***	18	
***Others***	12	
Total	1280	

*ESBL* extended-spectrum β-lactamase, *MRSA* methicillin-resistant S aureus, *VRE* vancomycin-resistant Enterococcus

*Vancomycin resistance was confirmed by Vitek 2C

#### Gram-negative isolates

Table 3 shows that the least frequency of resistance was recorded against nitrofurantoin (52.5%), amikacin (58.01%), followed by imipenem (59.78%) and meropenem (61.82%). Colistin was the most promising antibiotic as all *Acinetobacter* and *Pseudomonas* isolates showed 100% susceptibility  to it. Data also revealed that some antimicrobials showed a statistically significant difference in their antimicrobial activities to different bacterial isolates.

**Table 3:** Antibiotic resistance pattern of the prevalent gram-negative pathogens isolated from patients admitted in ICU, Ain Shams University Hospitals, Egypt, March 2018-February 2019

Antibiotic	Resistant isolates, *n* (%)						Total , *n*/*N*(%) *(N = 736)*	*p* value
	*Acinetobacter* spp*.* *(n = 128)*	*Klebsiella* spp *(n = 288)*	*E.coli* spp*.* *(n = 172)*	*Pseudomonas* spp*.* *(n = 114*	*Proteus* spp*.* *(n = 23*	*Enterobacter* spp. *(n = 11)*		
*Amikacin*	113 (88.28)	208 (72.22)	21 (12.21)	76 (66.66)	6 (26.08)	3 (27.27)	427/736 (58.01)	*p* < 0.001*
*Amoxicillin/clavulanate*	**NA	245 (85.06)	124 (72.09)	**NA	12 (52.17)	3 (27.27)	384/494 (77.73)	*p* < 0.001*
*Ampicillin/sulbactam*	110 (85.93)	277 (96.18)	155 (90.11)	**NA	14 (60.08)	4(36.36)	560/622 (90.03)	*p* < 0.001*
*Cefepime*	110 (85.93)	276 (96.83)	157 (91.27)	91 (79.82)	7 (30.43)	8 (72.72)	649/736 (88.17)	*p* < 0.001*
*Cefotaxime*	124 (96.87)	285 (98.95)	152 (88.37)	**NA	12 (52.17)	10 (90.90)	583/622 (93.72)	*p* < 0.001*
*Cefoxitin*	**NA	248 (86.11)	92 (53.48)	**NA	7 (30.43)	10 (90.90)	357/494 (72.26)	*p* < 0.001*
*Cefpodoxime*	**NA	282 (97.91)	160 (93.02)	**NA	10 (43.47)	9 (81.81)	461/494 (93.31)	*p* < 0.001*
*Ceftazidime*	115 (89.84)	279 (96.87)	156 (90.69)	92 (80.70)	8 (34.78)	10 (90.90)	660/736 (89.67)	*p* < 0.001*
*Ceftriaxone*	127 (99.21)	285 (98.95)	152 (88.37)	**NA	12 (52.17)	10 (90.90)	586/622 (94.21)	*p* < 0.001*
*Ciprofloxacin*	111 (86.71)	258 (89.58)	122 (70.93)	91 (79.82)	13 (56.52)	6 (54.54)	601/736 (81.65)	*p* < 0.001*
*Doxycycline*	85 (66.41)	235 (81.59)	123 (71.51)	**NA	19 (82.60)	9 (81.81)	471/622 (75.72)	0.008*
*Gentamicin*	97 (75.78)	204 (70.83)	66 (38.37)	81 (71.05)	10 (43.47)	5 (45.45)	463/736 (62.90)	*p* < 0.001*
*Imipenem*	101 (78.91)	225 (78.12)	32 (18.60)	75 (65.78)	4 (17.39)	3 (27.27)	440/736 (59.78)	*p* < 0.001*
*Levofloxacin*	109 (85.15)	231 (80.21)	119 (69.18)	87 (76.31)	14 (60.86)	5 (45.45)	565/736 (76.76)	0.001*
*Meropenem*	103 (80.46)	228 (79.16)	34 (19.76)	81 (71.05)	4 (17.39)	5 (45.45)	455/736 (61.82)	*p* < 0.001*
*Piperacillin/tazobactam*	107 (83.59)	250 (86.81)	95 (55.23)	80 (70.17)	5 (21.73)	10 (90.90)	547/736 (74.32)	*p* < 0.001*
*Tetracycline*	114 (89.06)	240 (83.33)	130 (75.58)	**NA	17 (73.91)	3 (27.27)	504/622 (81.02)	*p* < 0.001*
*Tobramycin*	90 (70.31)	234 (81.25)	106 (61.62)	92 (80.70)	6 (26.08)	7 (63.63)	535/736 (72.69)	*p* < 0.001*
*Trimethoprim/sulfamethoxazole*	87 (67.96)	241 (83.68)	137 (79.65)	**NA	16 (69.56)	3 (27.27)	484/622 (77.81)	*p* < 0.001*
*Colistin*	0 (0)	**NA	**NA	0 (0)	**NA	**NA	**NA	–
*Nitrofurantoin*	NA	77 (76.23)	32 (30.18)	**NA	**NA	0 (0)	109/209 (52.15)	*p* < 0.001*
No of urine samples	33	101	106	49	5	2	296	

**NA = not applicable

* significant at *p*<0.05

Table 3 also shows that among the obtained gram-negative pathogens, *Klebsiella* was the one that displayed the highest level of multidrug-resistance (87.84%) followed by *Acinetobacter* (83.59%). *E. coli* and *Pseudomonas* spp. showed almost identical levels of multidrug-resistance (73.68%, 72.02%) respectively. Although 70% or more of the *E. coli* isolates possessed resistance against the majority of the available antibiotic options, a few agents as amikacin, gentamicin, imipenem, meropenem, and nitrofurantoin showed potential antimicrobial activity with percentage of resistance of (12.21%, 38.37%, 18.60%, 19.76%, 30.18%), respectively.

**Table 4:** Antibiotic resistance pattern of the prevalent Gram-positive pathogens isolated from patients admitted in ICU, Ain Shams University Hospitals, Egypt, March 2018-February 2019

Antibiotic	Resistant isolates, *n* (%)			Total , *n*/*N*(%) *(N = 381)*	*p* value
	*Staphylococcus aureus* *(n = 69)*	***Coagulase-negative staphylococci*** *(n = 161)*	*Enterococcus* spp. *(n = 151)*		
*Amikacin*	34 (49.29)	61 (37.88)	**NA	95/230 (41.30)	0.108
*Ampicillin*	NA ***67	NA ***156	98 (64.90)	321/381 (84.25)	–
*Cefoxitin*	51 (73.91)	125 (77.63)	**NA	176/230 (76.52)	0.541
*Penicillin*	67 (97.10)	156 (96.89)	118 (78.14)	341/381 (89.50)	*p* < 0.001*
*Clindamycin*	36 (52.17)	97 (60.2)	**NA	133/230 (57.82)	0.256
*Erythromycin*	43 (62.31)	130 (80.74)	147 (62.31)	320/381 (83.98)	*p* < 0.001*
*Ciprofloxacin*	36 (52.17)	107 (66.45)	131 (86.75)	274/381 (71.91)	*p* < 0.00*1
*Doxycycline*	29 (42.02)	70 (43.47)	38 (25.16)	137/381 (35.95)	0.002*
*Gentamicin (low dose)*	51 (73.91)	98 (51.30)	**NA	149/230 (64.78)	0.058
*Gentamicin (high dose)*	**NA	**NA	95 (62.91)	95/151 (62.91)	–
*Linezolid*	0 (0)	0 (0)	0 (0)	0/381 (0)	–
*Levofloxacin*	33 (47.82)	97 (60.24)	115 (76.15)	245/381 (64.30)	0.001*
*Teicoplanin*	6 (8.69)	25 (15.52)	26 (17.21)	57/381 (14.96)	0.250
*Tetracycline*	38 (55.07)	76 (47.20)	53 (35.09)	167/381 (43.83)	0.011*
*Tobramycin*	40 (57.97)	117 (72.67)	**NA	157/230 (68.26	0.028*
*Trimethoprim/sulfamethoxazole*	10 (14.49)	100 (62.11)	**NA	110/230 (47.82)	*p* < 0.001*
*Nitrofurantoin*	0 (0)	4 (20.00)	38 (28.12)	42/99 (42.42)	0.021*
*Vancomycin*	0 (0)	0 (0)	10 (6.62)	10/381 (2.62)	–
No. of urine samples	5	20	74	99	

***The resistance for ampicillin was deduced from cefoxitin

**NA = not applicable

* significant at *p*<0.05

Both *Proteus* and *Enterobacter* spp. isolates showed the least antibiotic resistance among gram-negative pathogens. In *Enterobacter* isolates, the highest level of resistance was recorded against cephalosporins, piperacillin/tazobactam, doxycycline, and tobramycin in contrast to the rest of antimicrobials which still had considerable activity against *Enterobacter*. Proteus isolates had the maximum resistance levels observed towards ampicillin/sulbactam (60.08%), doxycycline (82.60%), levofloxacin (60.86%), tetracycline (73.91%), and trimethoprim/sulfamethoxazole (69.56%). Fortunately, the rest of the antibiotics still had a favorable potential against *Proteus*.

## Discussion

The excessive use of antibiotics has led to a vast widespread prevalence of antimicrobial resistance. As time passes, bacterial pathogens will defy every antibacterial option, thus, becoming extremely hard to control. Hence, the WHO identified it as an international health prime concern [12, 13].

To control this mounting predicament, comprehensive antibiotic stewardship in poor countries is fundamental. However, enough data regarding antimicrobial resistance are unavailable to precisely measure the extent of the problem. The few available studies concerning ICUs suggest that they are hotbeds of emerging high-level resistance. Hence, additional studies in other countries and healthcare settings are encouraged [14].

 In this study, gram-negative isolates (57.5%, *n* = 736) were more prevalent compared to gram-positive ones (31.1%, *n* = 399). Comparable results were found by Halim et al. [15] where gram-negative bacteria took the upper hand among all nosocomial pathogens (53%) while gram-positive organisms represented 37.9%. Similarly, gram-negative organisms constituted 65.7% of cases in a study conducted by Sawhney and colleagues [16].

Most of the isolates were recovered from blood cultures (44.84%) followed by urine (41.41%), unlike the results of Shebl and Mosaad who reported higher recovery from urine specimens in comparison with blood cultures [3].

Among gram-negative organisms, *Klebsiella*, represented the majority (22.5%) followed by *E. coli* (13.4%). On the other hand, CONS (12.5%) was the most common gram-positive pathogen.

 These results were similar to the results reported by other researchers studying bacterial strains in Egypt [15, 17] as well as from countries other than Egypt. Osifo, and Aghahowa from Nigeria reported that *E. coli* and *Klebsiella pneumoniae* were the most frequently isolated pathogens [18]. However, others reported a higher level of *E. coli* than *Klebsiella* spp. [3, 19] and *S. aureus* was much higher than CONS unlike our study [3]. The high prevalence of CONS was justified by Basiri and coworkers, who stated that it could be the overuse of invasive devices with repeated manipulation by healthcare workers and inadequate infection control measures [20].

As regards the distribution of pathogens among the different clinical specimens in the present work, CONS and *Klebsiella* spp. were the most frequently isolated from blood cultures. Man et al. from Romania also stated that CONS were the most commonly isolated pathogens from blood culture in their study; however, *E. coli* came before *Klebsiella* spp. as a cause of bacteremia [21].

In this study, *E. coli* and *Klebsiella* spp. were the main pathogens recovered from urine. Similarly, Duffa et al. [22] reported that *E. coli* and *Klebsiella* spp. were highly encountered pathogens in urine. As for the predominating pathogens in wound specimens, *Klebsiella*, *Pseudomonas*, and *Acinetobacter* spp*.* were highly recovered organisms in the current research. Ibrahim [23] from Saudi Arabia noticed that most the common wound pathogens were *Proteus* mirabilis followed by *Klebsiella pneumoniae*. However, in a research by Magdy and colleagues [24], *S. aureus* was the most common followed by *Pseudomonas aeruginosa*, *Klebsiella pneumoniae*, and *E. coli.*

The difference between the current study and other studies regarding type and frequency of pathogens could be linked to several factors like environmental conditions, health practices, patient conditions, personal hygiene, number of patients involved in each study, and laboratory procedures [3].

The highest percentage of resistance among gram-positive organisms was exhibited towards penicillin (89.5%) followed by erythromycin (83.98%) and cefoxitin (76.52%). Resistance to vancomycin was minimal (2.62%) among gram-positive organisms while no resistance was noted against linezolid (0%). Hove et al. [25] reported that the highest rates of resistance were observed against penicillin (90.0%) and oxacillin (64.0%). The overall resistance towards penicillin and cefoxitin among staphylococcal isolates of Magdy and colleagues [24] from Egypt was in agreement with the present study. However, their *S. aureus* isolates displayed higher resistance opposite to the current study where CONS was the species with higher resistance. Moreover, their results were much higher as regards vancomycin resistance which displayed resistance rates of 32.4% and 41.2% by *S. aureus* isolates and CONS respectively [24]. Results of Shebl and Mosaad [3] from Egypt were in contrast to the current study as regards vancomycin and linezolid with a resistance of 10.8% and 11.3%, respectively. However, the results of other researchers regarding vancomycin and linezolid were in favor to that obtained in the present study where Al-Zoubi and his colleagues reported that all their *S. aureus* isolates were 100% susceptible to vancomycin [26]. Also, Basak et al. [11] and Mahmoud et al. [27] reported 100% susceptibility to both vancomycin and linezolid. The higher antibiotic resistance rates reported by the above-mentioned researches in comparison to the current results might be attributable to inconvenient use of antimicrobials, geographic and socioeconomic variations, sampling biases, and dissimilar patients’ characteristics [28].

As regards *Enterococci*, the overall resistance pattern was comparable to that reported by Said and Abdelmegeed [29] with the exception of the higher linezolid resistance reported (9.7%) in their study. Likewise, the resistance pattern of ciprofloxacin, erythromycin, gentamicin, and linezolid agrees with that reported by Zalipour and coworkers [30]. Increased resistance to macrolides and quinolones among enterococcal clinical isolates might be attributed to their massive use for the treatment of enterococcal infections [31, 32].

In the present study, gram-negative isolates exhibited high resistance to almost all the used antibiotic classes with the least frequency recorded against nitrofurantoin (52.5%), amikacin (58.01%), followed by imipenem (59.78%) and meropenem (61.82%). All isolates of *Pseudomonas* and *Acinetobacter* showed 100% susceptibility to colistin so it can be considered a good therapeutic option [11]. These findings coincide well—with very few exceptions—with those of Eldomany and Abdelaziz [33] from Egypt who reported high antibiotic resistance against their tested isolates of *Acinetobacter*, *Pseudomonas*, *Klebsiella*, *Enterobacter*, and *E. coli* from cancer patients.

It is worthy to point to the fact that the high level of resistance observed among the gram-negative organisms in the current study agrees well with the results reported by other researchers [33–35]. Such elevated resistance in *Enterobacteriaceae* may be attributed to β-lactamase activity [36].

Generally, the resistance patterns of *Acinetobacter* and *Proteus* in the current research were in concordance with Ibrahim [23],  although *E. coli* isolates in Ibrahim’s study displayed similar results as regards amikacin, gentamycin, imipenem, and piperacillin/tazobactam but with much lower resistance for the rest of antibiotics. Regarding *Pseudomonas* and *Klebsiella*, both showed lower resistance rates in contrast to the present study [23]. Several causes can be responsible for the increased incidence of drug-resistance detected in the present study. The prime reason may be the common practice in Egypt where almost all patients—before hospital admission—take diverse antibiotics either prescribed by doctors or self-medication due to over-the-counter antibiotics administered mostly in an improper dose and for an inadequate period [37, 38]. Other potential causes are the geographical divergence as well as the genetic variations among pathogens from different studies. But unfortunately, data about the molecular characterization of the strains included in the current study are not available.

It is obvious that the obtained results in the current study reflected the high prevalence of multidrug resistance especially among gram-negative pathogens. *Klebsiella* displayed the highest degree of multidrug-resistance (87.84%) followed by *Acinetobacter* (83.59%). *E. coli* and *Pseudomonas* spp. showed almost identical levels of multidrug-resistance (73.68%, 72.02%) respectively, while *Proteus* and *Enterobacter* spp. isolates exhibited the lowest resistance among all the gram-negative pathogens.

There is a tremendous increase in MDR gram-negative bacteria in hospitals and especially in the intensive care units (ICU). Such resistance is most notable in ICUs due to the unrestrained usage of antibiotics in ICU in comparison to other hospital departments and most of these infections were caused by gram-negative bacilli [39].

It is important to note that the data presented in this study provide a general overview of the current horrifying situation in the hospital under study. This implicates that an action has to be taken to stop this catastrophe by starting an effective action plan for containment.

## Conclusions

The majority of the pathogens in the studied hospital have evolved resistance to most of the antibiotics. This foreshadows an inevitable catastrophe that threatens the future of the medical sector and requires special concern and continuous monitoring.

 Conducting more studies to obtain a complete picture of the local and international situation of antibiotic resistance is mandatory in order to guarantee effective antibiotic stewardship before reaching a deadlock with no way out.

## Data Availability

All data generated or analyzed during this study are included in this published article.

## Abbreviations

MDRO: Multidrug-resistant organisms
ASUH: Ain Shams University Hospitals
ICU: Intensive care unit
CDC: The Centers for Disease Control and Prevention
WHO: World Health Organization
CLSI: Clinical Laboratory Standards Institute
ESBL: Extended-spectrum beta-lactamase
MRSA: Methicillin-resistant *Staphylococcus aureus*
MR-CONS: Methicillin-resistant coagulase-negative
VRE: Vancomycin-resistant *Enterococci*
BHI: Brain heart infusion agar
VISA: Vancomycin-intermediate *Staphylococcus aureus*
VRSA: Vancomycin-resistant *Staphylococcus aureus*
*S. aureus*: *Staphylococcus aureus*
*E.coli*: *Escherichia coli*
